# Psychometric properties of the Slovenian version of Internet Disorder Scale–IDS-15

**DOI:** 10.1371/journal.pone.0276663

**Published:** 2022-10-21

**Authors:** Mark Žmavc, Halley M. Pontes, Mark D. Griffiths, Špela Selak

**Affiliations:** 1 National Institute of Public Health, Ljubljana, Slovenia; 2 Department of Organizational Psychology, Birkbeck, University of London, London, United Kingdom; 3 International Gaming Research Unit Psychology Department, Nottingham Trent University, Nottingham, United Kingdom; Aalborg University, DENMARK

## Abstract

**Background:**

Conceptualising internet addiction and assessing its symptoms has presented a significant challenge for researchers over the past 25 years. Recently, the Internet Disorder Scale (IDS-15), which is based on the criteria for Internet Gaming Disorder (IGD) from DSM-5, has emerged as a promising instrument to assess internet addiction. The main objective of the present study was to evaluate the psychometric properties of the Slovenian IDS-15.

**Methods:**

The sample was recruited from the National Survey on the Use of Tobacco, Alcohol and Other Drugs that was conducted in 2018 on a nationally representative sample (N  =  16,000; age range: 15–64 years; 62.4% response rate). The final sample comprised 9,161 participants, with 80.9% reporting having used the internet at least once a week (n  =  7,413). A structured questionnaire was designed and internet addiction was assessed using the IDS-15.

**Results:**

Confirmatory Factor Analysis showed acceptable fit to the proposed four-factor structure of the IDS-15. The reliability, and criterion, convergent and discriminant validity were also found to be adequate with a notable exception of the first item of the scale, as shown by its lower factor loading and higher variability. Additionally, latent profile analysis was used to distinguish between internet users with low (*n* = 3,818; 51.5%), medium (*n* = 3,111; 42.0%) and high (*n* = 484; 6.4%) addiction risk. Furthermore, the high-risk class was associated with higher IDS-15 factor scores, higher frequency of internet use in leisure time, and lower age of first internet use.

**Conclusions:**

The present study provides new insights about the strengths and shortcomings of the IDS-15. Moreover, the results provide an insight into the prevalence of internet addiction in Slovenia, as well as associations with other potential factors. The results serve as the basis for further analyses on internet addiction epidemiology, policymaking activities, and design for targeted public health interventions in Slovenia.

## Introduction

Excessive use of digital technologies and associated harm to users’ wellbeing has received significant attention from researchers over the past 25 years. More specifically, internet addiction (IA) has been a highly debated topic, lacking consensus on many of the core aspects of the concept. This is evident even at the terminological level, where ‘internet addiction disorder’ [1], ‘pathological internet use’ [2], ‘problematic internet use’ [3], and ‘internet use disorder’ [4] have been commonly used as alternatives to IA [5]. IA broadly refers to *“excessive or poorly controlled preoccupations*, *urges or behaviours regarding internet use that lead to impairment or distress”* [6]. The authors describing IA-related concepts have drawn from various theoretical foundations and empirical findings, such as the cognitive-behavioural approach [2], the components model of addiction [7], neuropsychological and neuroimaging findings [8], the DSM-IV criteria for gambling addiction [5], impulse-control disorders in general [3] or the DSM-5 criteria for Internet Gaming Disorder (IGD) [9].

According to Griffiths et al. [10], there is an important distinction between addiction *to* the internet and addictions *on* the internet. Here the authors assert that excessively using the internet as a medium to access particular addictive content does not equate to being addicted to the internet [11, 12]. On the other hand, Király et al. [13] argued that the internet is often more than a medium and it significantly changes aspects of the original activity. For example, online gaming has a stronger social dimension (and consequently, a stronger pull) than offline gaming due to social interactions between players. For some users, the internet as a whole (through anonymity, diversity of content, interactivity and accessibility) provides an environment that consistently facilitates feelings of belongingness, which can be highly psychologically and physiologically rewarding [10]. The same could be argued for feelings of autonomy and competence, two other basic psychological needs [14]. Davis [2] distinguished between specific and generalised Pathological Internet Use (PIU), describing the latter as a general, multidimensional overuse of the internet, which may include wasting time without a clear objective. He asserted that the pathology of individuals with generalised PIU would likely not even exist in the absence of the internet. The dichotomy of generalised versus specific PIU has been supported by many researchers (e.g. [15, 16]). In a recent literature review, Kamolthip et al. [17] concluded that these conditions require different treatments and should be evaluated independently.

Moreover, while many recent conceptualisations of PIU have focused on specific PIU, such as social media addiction [18], IGD [19], internet gambling addiction [20] and internet pornography addiction [21], research efforts towards a generalised PIU concept are arguably equally important [22]. Furthermore, the emerging and closely related concept of smartphone addiction should also be noted. Montag et al. [4] argued that devices such as smartphones are characterised by specific behavioural usage patterns and technological features, and that a particular device may be preferred for a specific (type of) online activity. Following this logic, they propose the following dichotomy; PIU predominantly mobile and PIU predominantly non-mobile.

The lack of consensus in the conceptual background of IA has also been evident from the various attempts to assess the phenomenon. Methodological strengths and shortcomings of existing instruments assessing IA have been described in a review by Király et al. [23], where inconsistencies between instruments have been observed at many levels (i.e., theoretical basis, factor structures and psychometric properties). This led Pontes and Griffiths [24] to develop a new psychometric tool (i.e., Internet Disorder Scale; IDS-15) by adapting the DSM-5 criteria IGD [19] to that of IA. The IDS-15 contains 15 items, defining four qualitatively distinct IA-related domains: (i) escapism and dysfunctional emotional coping, (ii) withdrawal symptoms, (iii) impairments and dysfunctional self-regulation, and (iv) dysfunctional internet-related self-control.

To date, three IDS-15 validation studies have been published. The first IDS-15 validation study by Pontes and Griffiths [24] included 1,094 participants aged from 16 to 70 years old (Mean_age_ = 33 years) and collected the data utilising opportunistic sampling. The scale demonstrated acceptable factorial, convergent, and discriminant validity. The first-order Confirmatory Factor Analysis (CFA) including its four-factor model showed adequate fit to the data. Internal reliability estimates for the four dimensions of IDS-15 ranged from .79 to .85. The overall IDS-15 score was positively associated with weekly time spent on the internet, age of first internet use, and relationship status. Additionally, high addiction risk was negatively associated with age and positively associated with frequency of cigarette smoking while drinking alcohol frequency was unrelated to IA risk.

The Italian IDS-15 validation study [25] included 1,094 participants aged from 16 to 70 years old (Mean_age_ = 33 years) and collected the data utilising opportunistic sampling. The scale demonstrated adequate construct validity through a second-order CFA and showed acceptable indices for both first- and second-order structural models. Criteria for satisfying convergent and discriminant validity were also met. Internal reliability estimates ranged from .76 to .92 for each dimension of the IDS-15. The analyses also supported measurement invariance of the model across genders. Furthermore, positive correlations were observed between IDS-15 scores, IGD, and social media addiction.

Similar to the previous studies, the Persian IDS-15 validation study [26] included 1,272 high-school students (Mean_age_ = 15.5 years) and collected the data utilising a multistage randomised sampling process. The scale demonstrated acceptable construct validity based on the indices derived from a second-order CFA. The authors reported excellent fit of all items using Rasch analyses. Internal reliability for scale dimensions was sufficient (.76-.90), as was the test-retest reliability of each item (.70-.88). The study found that being female and older were among the factors associated with IA.

The three IDS-15 validation studies provide a solid starting point towards establishing IDS-15 as a consistently reliable and valid tool to assess IA based on the DSM-5 framework. However, Koronczai et al. [27] proposed six criteria which any suitable IA instrument should meet: comprehensiveness, brevity, reliability and validity for different genders, age groups, cultures, and validation on clinical samples. The current empirical evidence for IDS-15 is still lacking for criteria 4–6 because the validity for various age groups and cultures has yet to be established, and showing how the IDS-15 score relates to clinical diagnoses of IA is still required. Therefore, the aim of the present study was to provide further evidence for the reliability and validity across genders and across cultures using a large representative dataset from a national survey conducted in Slovenia. Additionally, the study explicitly tested whether the proposed factor structure model for the IDS-15 fitted the data across various age groups. Following the example of the validation studies summarised above, users of varying risks for IA were differentiated using latent profile analysis (LPA). The intention was to obtain an approximate estimate of addicted internet users and to aid future efforts for determining a cut-off score for IA on the IDS-15. Lastly, the study calculated the correlations between IDS-15 scores (as well as risk category) and sociodemographic characteristics, gaming, gambling, and drug, alcohol and tobacco use.

## Methods

### Participants

The sample for the IDS-15 validation study comprised 7,413 participants, all of who reported using the internet at least once a week (47.0% males, n = 3,484, and 53.0% females, n = 3,929). Participants’ age ranged from 15 to 64 years, with an average age of 39.2 years (SD = 13.7 years). Education levels also varied, with 11.8% (n = 875) reported having achieved primary or lower education, 50.4% (n = 3,736) secondary education, and 37.8% (n = 2,802) tertiary education. In terms of employment status, 67.7% (n = 5,019) were employed, 18.3% (n = 1,357) were students, 6.9% (n = 511) were unemployed and 7.1% (n = 526) were retired. Each of the 12 Slovenian regions was represented in the sample, although more than a quarter of participants lived in the Central Slovenia Statistical Region.

### Procedure

#### Sampling

The sample was recruited from the National Survey on the Use of Tobacco, Alcohol and Other Drugs that was conducted in 2018, in collaboration with the Statistical Office of Slovenia. A total of 16,000 inhabitants of Slovenia living in private (not institutionalised) households (age range: 15–64 years) were invited to participate (8,000 in the spring and 8,000 in the autumn of 2018). The final sample comprised 9,161 participants (62.4% response rate). A two-stage sampling explicitly stratified by size and type of settlement and implicitly by statistical regions was carried out. Data was collected using a mixed method via Computer-Assisted Web Interviewing (CAWI) and Computer-Assisted Personal Interviewing (CAPI). Namely, all individuals selected in the sample received a notification letter inviting them to complete the survey over the web. In the letter, they were informed about the voluntary participation in the survey, about the purpose and procedure of the survey, and assured that their responses would be confidential and carefully protected. By continuing with the survey questions, they consented to participate in the study. The web survey was available online throughout the data collection period. Individuals who did not respond were additionally contacted by an interviewer and invited to participate in the study. Following their agreement to participate, the data collection took place in their homes using computer-assisted face-to-face interviews. Thorough instructions for survey application were given to the interviewers. The structured questionnaire was designed for data collection using the methodology of the European Monitoring Centre for Drugs and Drug Addiction (EMCDDA) to ensure comparability with other studies within the European Union. Additionally, assessment tools for IA, gaming, and gambling were included in the survey. IA was assessed using the IDS-15. A majority of the participants (80.9%) reported using the internet at least once a week (n  =  7,413).

#### Adaptation of the Slovenian IDS-15

The IDS-15 was translated from the original English version into Slovenian by two experts in the field of digital addiction. This was then additionally checked by the lead methodologists of the research project (experts with a background in psychology, psychometrics, and sociology and with many years of experience in coordinating the implementation of national surveys in public health). The translation process followed the three-step Eurostat translation protocol [28]. No adaptations due to cultural differences were deemed necessary.

### Measures

#### Internet addiction

The IDS-15 is a psychometric tool, which assesses the severity of IA among individuals, based on APA framework for IGD proposed in the DSM-5 [24]. The items refer to users’ online leisure activity, excluding internet use for work or school, from any device with internet access in the past 12 months. The IDS-15 contains 15 items comprising four IA-related domains: Escapism and Dysfunctional Emotional Coping (EDEC), Withdrawal Symptoms (WS), Impairments and Dysfunctional Self-Regulation (IDSR), and Dysfunctional Internet-related Self-Control (DISC). Participants rate items using a five-point Likert Scale, from 1 (“Strongly disagree”) to 5 (“Strongly agree”). Total scores range from 15 to 75, with higher scores indicating higher severity or higher risk of IA. The psychometric properties of the Slovenian IDS-15 are reported in the Results section.

#### Gaming disorder

The Internet Gaming Disorder Scale (IGDS9-SF) [29], a nine-item screening tool, was used to assess the severity of IGD and its harmful effects by examining online and offline gaming over the past 12 months. The items (e.g., *“Do you systematically fail when trying to control or cease your gaming activity*?*”*) are answered using a five-point Likert scale from 1 (“Never”) to 5 (“Very often”). Total scores range from 9 to 45, with higher scores indicating higher severity of IGD [30, 31]. The IGDS9-SF is an established cross-culturally validated measure to assess IGD [30, 31]. The Slovenian version was adapted and validated by Pontes et al. [32] in a study on IGD among Slovenian youth. Cronbach’s α of the Slovenian IGDS9-SF in the present study was .89.

#### Gambling disorder

The Berlin Inventory of Gambling Behavior–Screening (BIG-S) [33], a reliable and valid screening tool, was used to assess gambling addiction. The inventory consists of 14 questions, which represent an operationalisation of the ten DSM-5 criteria for gambling disorder. The items (e.g., *“Have you repeatedly spent more time or money on gambling than originally intended*?*”*) are answered in a dichotomous format (yes/no) and refer to gambling-related behaviour throughout the lifespan. Total score ranges from 0 to 10, with a score of 5 and above being indicative of gambling disorder. The Slovenian BIG-S was adapted according to the same protocol as the IDS-15 (see ‘Adaptation of the Slovenian IDS-15’). Cronbach’s α of the Slovenian BIG-S was .81.

#### Other measures

Additionally, participants were asked about the frequency of their alcohol, tobacco, and illegal drug use. Lastly, key demographic characteristics were also reported, such as gender, age, level of education, employment status, region, and monthly income.

### Statistical analysis

The statistical methods used comprised the following; (i) frequencies and descriptive statistics of key variables, (ii) CFA using robust maximum likelihood estimation (MLR) for evaluating construct validity, along with average variance extracted (AVE) and maximum shared variance (MSV) indices for determining convergent and discriminant validity, (iii) CFA for multiple groups to evaluate measurement invariance between genders and age groups (iv) internal consistency and composite reliability (CR) indices for purposes of reliability estimation, (v) bootstrapped correlation matrix with the associated bias-corrected accelerated 95% confidence interval for evaluating criterion validity, and (vi) LPA with associated fit indices (Aikake Information Criterion—AIC, Bayesian Information Criterion—BIC, Bootstrapped Likelihood Ratio Test—BLRT *p*-value, and entropy) to identify latent groups of internet users. LPA is a statistical technique used to identify groups of individuals with similar patterns of scores in any set of variables [34].

To evaluate the model fit given by CFA, standard thresholds for acceptable fit were referred to (comparative fit index (CFI) > .90, Tucker-Lewis index (TLI) > .90, root mean square error of approximation (RMSEA) < .10, standardised root mean square residual (SRMR) < .10) and good fit (CFI > .95, TLI > .95, SRMR < .08, RMSEA < .08) as proposed by Hooper et al. [35]. The obtained AVE and MSV values were evaluated based on suggestions of Hair et al. [36]. Lastly, to assess measurement invariance, the fit of Model 1 was evaluated with groups based on gender and age, with constrained factor structure (and loadings, intercepts) across groups. Sufficient configural (and metric, scalar) invariance was concluded, if the resulting changes in CFI, RMSEA and SRMR fit indices were within the thresholds recommended by Chen [37] (ΔCFI < .010, ΔRMSEA < .015, ΔSRMR < .010). All statistical procedures were performed with the R software environment for statistical computing and graphics, version 1.4.1717 [38], using “semTools” [39], “lavaan” [40] and “tidyLPA” [41] statistical packages.

### Ethical considerations

This study uses data from the National Survey on the Use of Tobacco, Alcohol and Other Drugs 2018, conducted by Slovenian National Institute of Public Health, which is an authorised producer of national health and health care statistics (playing a role of other national authority in the official statistics). The legal framework for this survey was set in the National Statistics Act and Annual Programme of Statistical Surveys for 2018 which was approved by the Information Commissioner of Slovenia. Thus, since all national health and healthcare statistics are carried out in the same manner, no Institutional review board or ethical committee was needed to additionally approve or waive the study.

## Results

### Descriptive statistics

Table 1 shows descriptive statistics of the total IDS-15 score and the average item score for each of its four dimensions in the overall sample. Additionally, weekly internet use in leisure time was reported: less than seven hours weekly (n = 4,448, 60.0% of users), 8–14 hours (n = 1,542, 20.8%), 15–20 hours (n = 556, 7.5%), 21–30 hours (n = 378, 5.1%), 31–40 hours (n = 193, 2.6%), and more than 40 hours (n = 296, 4.0%).

**Table 1:** Descriptive statistics of IDS-15 score and its dimensions.

	Mean (SD)		
Variable	Total	Males	Females
IDS-15 total	28.78 (8.88)	29.12 (8.70)	28.48 (9.04)
EDEC	2.26 (0.81)	2.26 (0.79)	2.25 (0.82)
WS	1.60 (0.67)	1.63 (0.67)	1.57 (0.65)
IDSR	1.80 (0.78)	1.82 (0.77)	1.78 (0.79)
DISC	2.05 (0.83)	2.09 (0.82)	2.02 (0.83)

EDEC = Escapism and Dysfunctional Emotional Coping; WS = Withdrawal Symptoms; IDSR = Impairments and Dysfunctional Self-Regulation, DISC = Dysfunctional Internet-related Self-Control

### Reliability

Reliability of the scale, calculated with McDonald’s ω, was .94. Table 2 shows the reliability coefficients (Cronbach’s α, McDonald’s ω, and composite reliability) for each of the four factors of the IDS-15. All coefficients indicated adequate internal reliability (> .70) of the IDS-15 factors, although EDEC was the least reliable of the four.

**Table 2:** Reliability coefficients of IDS-15 for each dimension.

	Cronbach’s α	McDonald’s ω	CR
EDEC	.73	.78	.78
WS	.91	.91	.91
IDSR	.87	.87	.87
DISC	.87	.88	.89

CR = composite reliability; EDEC = Escapism and Dysfunctional Emotional Coping; WS = Withdrawal Symptoms; IDSR = Impairments and Dysfunctional Self-Regulation, DISC = Dysfunctional Internet-related Self-Control

### Factorial validity

CFA was performed to assess the validity of the proposed four-factor structure of the IDS-15 (i.e., Model 1). Additionally, the analysis evaluated the fit of a one-factor model comprising all 15 items (i.e., Model 2). Table 3 shows the goodness of fit indices, as well as the models’ overall evaluation (see ‘Statistical analysis’). Model 1 provided the best (although still only acceptable) fit to the data. Factor loadings of all items (see Table 6) were statistically significant. The poor fit of Model 2 suggests that the latent structure of IA as assessed by the IDS-15 is likely not unidimensional.

**Table 3:** Goodness-of-fit indices and evaluations for the specified models.

	χ^2^	df	*p*-value	CFI	TLI	RMSEA	SRMR	Overall evaluation
Model 1	2298.6	84	< .001	.939	.924	.060	.037	Acceptable fit
Model 2	14719.8	90	< .001	.596	.529	.148	.112	Poor fit
Measurement invariance	Gender	Equal structure		.952	.940	.059	.035	Acceptable MI
		Equal loadings		.951	.943	.058	.038	Acceptable MI
		Equal intercepts		.950	.945	.057	.038	Acceptable MI
	Age group	Equal structure		.951	.939	.060	.040	Acceptable MI
		Equal loadings		.948	.941	.059	.044	Acceptable MI
		Equal intercepts		.936	.934	.062	.048	MI not established

CFI = comparative fit index; TLI = Tucker-Lewis index; RMSEA = root mean square error of approximation; SRMR = standardised root mean square residual; MI = measurement invariance

For the purposes of establishing whether the IDS-15 assesses the same construct similarly across different subgroups (i.e., measurement invariance), CFA for multiple groups was utilised (using Model 1). In the case of gender, the changes of CFI, RMSEA and SRMR fit indices when constraining factor structure, loadings or intercepts (Table 3), were within recommended thresholds (see ‘Statistical analysis’). When inspecting measurement invariance across age groups, the changes of fit indices were only acceptable when constraining factor structure and loadings, but not intercepts (i.e., CFI > .010)

### Convergent and discriminant validity

Convergent validity, shown by AVE values (see Table 4) over .50 is somewhat questionable in the case of the EDEC factor, while being satisfactory for the remaining latent factors. Discriminant validity of the factors was acceptable, as evidenced by values of MSV, which were all lower than AVE values for each factor. As an additional indication of discriminant validity, the square roots of AVE for each factor (i.e., bold diagonal values in Table 4) were higher than its correlation with other factors.

**Table 4:** Indices of reliability, convergent and discriminant validity for the four factors of IDS-15.

	Index		Correlations			
	AVE	MSV	EDEC	WS	IDSR	DISC
EDEC	.43	.28	**.66**			
WS	.72	.40	.53	**.85**		
IDSR	.63	.40	.48	.63	**.80**	
DISC	.69	.15	.34	.37	.39	**.83**

AVE = average variance extracted; MSV = maximum shared variance; EDEC = Escapism and Dysfunctional Emotional Coping; WS = Withdrawal Symptoms; IDSR = Impairments and Dysfunctional Self-Regulation, DISC = Dysfunctional Internet-related Self-Control

### Criterion validity

Table 5 shows the correlations between each IDS-15 dimension, total score or IA risk class (see ‘Latent profile analysis’ below), and key demographic or criterion variables. Importantly, IDS-15 dimensions show positive correlations with the IGD (*r* > .20), positive correlations with hours of weekly internet use (*r* > .20) and negative correlations with the age of first internet use (*r* < -.15). These are interpreted as support to the criterion validity of the IDS-15. The IDS-15 dimension scores were mostly unrelated to tobacco and alcohol use but showed low but significant negative correlations with illegal drug use. Additionally, dimension scores were negatively correlated with pathological gambling behaviour. In terms of demographic characteristics, individuals’ age was a strong predictor of high IDS-15 scores, with younger participants being at more risk. Lower education was a risk factor for the EDEC and WS dimensions but not for the IDSR and DISC dimensions. Dimension correlations with gender were relatively small, although male internet users had slightly higher IDS-15 scores. Lastly, monthly income was mostly unrelated to IDS-15 scores.

**Table 5:** Bootstrapped correlation matrix between IDS-15 (and its dimensions) and key demographic and criterion variables.

	Gender	Age	Education	Monthly income	Internet hours per week	Age of first internet use	IGDS	BIG-S	Tobacco use	Alcohol use	Illegal drug use
IA class	-.04**	-.18**	-.08**	.00	.23**	-.16**	.37**	-.17**	.00	-.01	-.05**
EDEC	-.00	-.33**	-.09**	.00	.35**	-.28**	.39**	-.22**	-.01	-.01	-.06**
WS	-.04**	-.19**	-.08**	.01	.24**	-.16**	.40**	-.18**	.00	-.01	-.04**
IDSR	-.03	-.23**	-.02	.02	.26**	-.21**	.45**	-.21**	-.06**	-.03	-.05**
DISC	-.04**	-.19**	.01	.04**	.22**	-.19**	.23**	-.13**	-.01	-.04**	-.06**
IDS-15	-.04**	-.31**	-.06**	.02	.35**	-.28**	.50**	-.25**	-.03	-.03	-.07**

**p < .01; IGDS = Internet Gaming Disorder Scale; BIG-S = Berlin Inventory of Gambling Behaviour–Screening; EDEC = Escapism and Dysfunctional Emotional Coping; WS = Withdrawal Symptoms; IDSR = Impairments and Dysfunctional Self-Regulation, DISC = Dysfunctional Internet-related Self-Control

### Item-level analysis

Psychometric analysis at the item-level is presented in Table 6. The factor loadings of items obtained from the CFA (Model 1) were generally satisfactory, except in the case of item EDEC-1 (i.e., *“I never go online to feel better”*). Consequently, the deletion of this item resulted in a significant improvement of EDEC factor reliability (+ 0.09). EDEC-1 also had the highest standard deviation and mean among all items of the IDS-15. Additionally, items EDEC-2 (i.e., *“I think that being online can greatly change my mood for the better”*) and DISC-1 (i.e. *“I could easily stop spending time online if I wanted to without any problem”*) displayed slightly higher standard deviation, mean and lower factor loadings compared to other items. DISC-1 was also the only other item besides EDEC-1 whose deletion improved the corresponding factor reliability.

**Table 6:** Descriptive and psychometric characteristics of IDS-15 at the item level.

		Mean	SD	Loading	Item-total corr.	Alpha if item deleted	Alpha of factor
EDEC-1 (R)	I never go online to feel better.	2.71	1.27	.31	.63	.82	.73
EDEC-2	I think that being online can greatly change my mood for the better.	2.38	1.13	.68	.78	.64	.73
EDEC-3	I go online to help me cope with any bad feelings I might have.	1.97	0.94	.84	.81	.60	.73
EDEC-4	I go online to forget about whatever’s bothering me.	1.97	0.98	.84	.81	.60	.73
WS-1	When I am not online I feel irritable, restless, anxious and/or frustrated.	1.53	0.69	.76	.83	.90	.91
WS-2	I feel sad if I am not able to go online.	1.67	0.82	.83	.88	.88	.91
WS-3	I tend to get anxious if I can’t check what’s happening online for any reason.	1.61	0.76	.89	.91	.86	.91
WS-4	I feel restless every time I am unable to go online.	1.58	0.74	.89	.91	.86	.91
IDSR-1	I think the amount of time I spend online has jeopardized the relationship with my partner.	1.76	0.93	.72	.82	.85	.87
IDSR-2	I think the amount of time I spend online is negatively impacting on important areas of my life.	1.77	0.90	.80	.87	.82	.87
IDSR-3	I would like to cut down the amount of time I spend online but it’s difficult for me to do.	1.90	0.96	.84	.87	.83	.87
IDSR-4	I often try to spend less time online but find I cannot.	1.78	0.88	.81	.83	.84	.87
DISC-1 (R)	I could easily stop spending time online if I wanted to without any problem.	2.26	1.07	.71	.87	.90	.87
DISC-2 (R)	I can easily cut down the time I spend online any time that I want to.	2.01	0.91	.96	.93	.74	.87
DISC-3 (R)	I am able to control and/or reduce the time I spend online.	1.88	0.83	.87	.89	.80	.87

EDEC = Escapism and Dysfunctional Emotional Coping; WS = Withdrawal Symptoms; IDSR = Impairments and Dysfunctional Self-Regulation, DISC = Dysfunctional Internet-related Self-Control

### Latent profile analysis

In order to describe various types of internet users based on their risk for IA, LPA was performed. The optimal number of latent classes was determined based on multiple indices, presented in Table 7. The AIC and BIC values were decreasing up to the 10-class solution, and the associated BLRT *p-*value was within statistical significance for up to seven classes (presumably due to the larger sample size). Due to relatively low classification certainty as well as difficulty of interpretation of this many latent classes, we opted to compare the class solutions based on the entropy criterion (a measure of classification certainty, where 1.00 represents complete certainty). Based on the highest entropy values, as well as highest minimum and maximum average latent class probability (Min. prob., Max. prob.), the three-class solution was ultimately recognised as optimal. In line with previous IDS-15 validation studies, these categories were labelled as “low addiction risk” (*n* = 3,818; 51.5%), “medium addiction risk” (*n* = 3,111; 42.0%), and “high addiction risk” (*n* = 484; 6.4%).

**Table 7:** Indices of classification quality for two to five class solutions, based on latent profile analysis.

Number of classes	AIC	BIC	Entropy	Min. prob.	Max. Prob.	BLRT (*p* value)
2	62000.72	62090.56	.78	.93	.95	< .01
3	56657.88	56782.28	.97	.97	.99	< .01
4	55344.36	55503.31	.94	.78	.98	< .01
5	54160.81	54354.31	.95	.78	.98	< .01

AIC = Aikake information criterion; BIC = Bayesian information criterion; BLRT = Bootstrapped likelihood ratio test

Characteristics of each IA class are shown in Table 8. High-risk users show higher scores across all IDS-15 dimensions. Notably, risk classes are particularly distinguished in terms of their WS factor score. In addition, significantly more high-risk users reported using the internet for at least 20 hours per week in leisure time, compared to the other two classes. Lastly, higher IA risk class corresponds with lower age of first internet use.

**Table 8:** IDS-15 dimension scores, weekly internet use and age of first internet use for each IA risk class.

	IA class		
	Low-risk	Medium-risk	High risk
EDEC	1.89	2.55	3.28
WS	1.06	2.01	3.22
IDSR	1.35	2.18	2.93
DISC	1.77	2.28	2.77
Weekly internet use (20+ hours)	7.3%	13.2%	36.1%
Age of first Internet use	26.25 years	23.62 years	18.29 years

EDEC = Escapism and Dysfunctional Emotional Coping; WS = Withdrawal Symptoms; IDSR = Impairments and Dysfunctional Self-Regulation, DISC = Dysfunctional Internet-related Self-Control

## Discussion

The aim of the present study was to continue the efforts of evaluating the psychometric properties of the IDS-15, previously done by Pontes and Griffiths [24], Monacis et al. [25], and Lin et al. [26]. More specifically, the study developed and evaluated the Slovenian IDS-15 on a large and nationally representative sample of Slovenian internet users (aged 15–64 years). Firstly, the reliability analysis showed that the Slovenian IDS-15 is a reliable instrument, both on the level of the scale (ω = .94) and its four sub-factors (α > .70). Furthermore, the CFA results indicated that the four-factor structure proposed by Pontes and Griffiths [24], sufficiently fitted the data. However, importantly, the factor loading of Item EDEC-1 was noticeably lower compared to other items, despite its statistical significance. Its (relatively) high variance, mean, and alpha-if-item-deleted also indicated there is room for improvement. On the other hand, the discriminant validity of the scale was adequate, which means that the IDS-15 factors assess distinct constructs pertaining to IA. On the final indicator of construct validity (i.e., measurement invariance), the present study confirmed the findings of Monacis et al. [25] and Lin et al. [26] regarding measurement invariance across both genders on a configural, metric and scalar level. Moreover, measurement invariance across age groups was established on a configural and metric (but not scalar) level in the present sample.

The LPA identified three latent profiles of internet users with regard to their risk of IA. A large majority of participants were classified as either low addiction risk (51.5%) or medium addiction risk users (42.0%), whereas only 6.4% of users were recognised to have a (comparatively) high-risk for IA. This figure is similar to the global prevalence rate of IA reported in a previous meta-analysis study [42], and a more recent one by Pan et al. (7.0%) [43]. The proportion of high-risk users was significantly higher in the three previous validation studies [24–26], which reported values between 26% and 42%. This discrepancy could be attributed to either the lower age of participants (adolescents and students) or the opportunistic sampling in the previous studies. Furthermore, individuals with higher addiction risk (as well as IDS-15 score and factor scores) obtained higher scores on all IDS-15 factors, were more likely to spend more time on the internet for leisure purposes, were younger, started using the internet earlier, and had a higher IGD score, which is consistent with the findings of Pontes and Griffiths [24]. Additionally, these results are comparable with the findings of a study on problematic internet use of Slovenian adolescents by Macur and Pontes [44], where it was reported more withdrawal symptoms (factor Obsession), lack of control (factor Control Disorder), and impairments (factor Neglect) among “high PIU risk” individuals.

Criterion validity analysis further showed that effects of gender, level of education, and monthly income were weak and inconsistent across IDS-15 factors, indicating that IA is not restricted to specific demographic groups. Frequencies of tobacco and alcohol use were not associated with IDS-15 scores. Furthermore, individuals reporting problematic gambling behaviour and illegal drug use were even less likely to obtain high IDS-15 scores. These findings strengthen the case of IA being a completely separate disorder to substance (and gambling) addictions. Based on the results above, the IDS-15 has adequate criterion validity.

The undesirable performance of item EDEC-1 (i.e., *“I never go online to feel better”*) may be due to two potential reasons. First, the wording of the item, specifically the use of the word *“never”*. For the average internet user, who has been accessing internet daily for multiple years, it would be very difficult to claim they have *“never”* gone online to feel better. The tendency to make such a claim, may be, at least in part, due to factors unrelated to internet use. Moreover, items using *“never”* and *“always”* are common in social desirability scales (i.e., [45]). The possibility that social desirability may be responsible for a non-negligible share of the variance of EDEC-1 should therefore be considered. A second potential reason for the subpar performance of EDEC-1 may be due to the reversed item effect, since EDEC-1 is the only reversed item in that factor (for an elaboration of the reversed item method bias, see [46]). Considering the apparent lack of observed issues with EDEC-1 in previous validation studies, the item translation was re-examined, but no obvious improvements could be made. Therefore, it is suggested that there be an alternative (non-reversed) item wording which would address both criticisms: *“I often go online to feel better”*.

An additional note related to the practice of using reversed items should be mentioned in the case of the IDS-15. The fourth IDS-15 factor, Dysfunctional Internet-related Self-Control (DISC) consists entirely of reversed items. The proposed grouping of these items, indicated by CFA, may not only be due to their content, but perhaps also due to their wording direction. Looking more closely, Items IDSR-3 and IDSR-4 target an ability, very alike to the one targeted by the DISC items (i.e., the ability to control/regulate time spent on the internet). Since the discriminant validity of the DISC factor seems completely sufficient, the question of which unique tendencies are assessed by the factor in question (as opposed to IDSR) should be further explored.

Since the publication of the first IDS-15 validation study, the criteria for Gaming Disorder (GD) have been introduced in the ICD-11 [47], and supported by a consensus of experts [48]. Importantly, escapism/mood regulation and withdrawal symptoms (two IDS-15 factors) are not used to assess GD in the ICD-11. Since the IDS-15 is partly based on the DSM-5 criteria for IGD, it should be explored to what extent these changes also apply to IA. Lastly, it was expected that both individuals with generalised PIU and specific PIU would obtain a high IDS-15 scores. It is recommended the scale application to be supplemented with individual interviews and questions regarding the type and frequency of their online activities to differentiate between the two PIU types. Additionally, questions regarding devices used can help to differentiate between PIU predominantly mobile and PIU predominantly non-mobile, as suggested by Montag et al. [4].

### Study limitations

Despite careful planning, design and implementation of the study as well as the sample population being representative, there are some limitations that should be mentioned. First, there is a possibility of bias associated with the response rate. Heavy internet users may have been more likely to decline their participation in a lengthy survey, due to their preoccupation with online activities or their general preference for engaging, online content. This could have led to underestimating the extent of IA symptoms in the population. Second, the absence of cognitive interviews during the adaptation process of the Slovenian IDS-15 may mean there is room for improvement in terms of item wording. Third, all the data were based on self-reports which are subject to well established method biases.

## Conclusions

The results of the present study contribute to the scientific literature concerning the validity of IDS-15. The study provided the data from the Slovenian version of IDS-15 scale, which add to the cultural variability of currently validated versions of the IDS-15 (i.e., English, Italian, Persian). To add to previous validation attempts of the IDS-15, the present study obtained a larger and more nationally representative sample of internet users between the ages of 15 and 64 years. The procedures and methods adopted to evaluate psychometric properties of previous versions of the IDS-15 were followed in the present study, with the addition of measurement invariance analysis across different age groups. The findings illustrated strengths and potential shortcomings of the IDS-15. The reliability and validity of the Slovenian IDS-15 and its factors were in general satisfactory. However, due to the concerns related to Item EDEC-1, testing its recommended reworded version would be sensible. Nonetheless, the results of the present study valuably contribute to the field of digital addictions. Namely, they provide an insight in the prevalence of IA in Slovenia, as well as associations with other factors that may help to explain this phenomenon. Therefore, the results reported serve as the basis for further analyses on IA epidemiology, policymaking activities, and design for targeted public health interventions in Slovenia, as well provide a basis for future studies. Future studies of the IDS-15 would make a significant contribution by addressing any of the issues raised in the Discussion, by further testing either formulation of Item EDEC-1, employing qualitative approaches to assess validity, establishing measurement invariance beyond gender, and obtaining data from a clinical sample.
